# Reverse aqua pump vacuum-assisted closure alternative and affordable solution for emerging cost in wound therapy: A case series

**DOI:** 10.1016/j.ijscr.2025.111981

**Published:** 2025-09-23

**Authors:** Meirizal, Rahadyan Magetsari, Mohammad Rizal Chaidir, Sumadi Lukman Anwar, Agung Susilo Lo, I. Made Dolly

**Affiliations:** a Doctoral Program in Medicine and Health Science, Faculty of Medicine, Public Health and Nursing, Universitas Gadjah Mada, Yogyakarta, Indonesia; b Orthopedics and Traumatology Department, RSUP Dr. Sardjito Hospital, Universitas Gadjah Mada, Yogyakarta, Indonesia; c Hand and Microsurgery Division, Orthopedics and Traumatology Department, RSUP Dr. Hasan Sadikin Hospital, Universitas Padjajaran, Bandung, Indonesia; d Oncology Surgery Division, Surgery Department, RSUP Dr. Sardjito Hospital, Universitas Gadjah Mada, Yogyakarta, Indonesia; e Faculty of Medicine, Public Health and Nursing, Universitas Gadjah Mada, Jl. Farmako, Sendowo, SekipUtara, Sleman, 55281, D.I.Yogyakarta, Indonesia

**Keywords:** NPWT, VAC, Innovation, Low-cost, Wound, Case series

## Abstract

**Introduction and importance:**

Soft tissue defects managed with conventional wound therapies are often inadequate. Vacuum-Assisted Closure (VAC) has proven to be an effective method for discharge removal, edema reduction, perfusion enhancement, and granulation tissue stimulation. This study assesses the novel Reverse Aqua Pump Vacuum-Assisted Closure (RAP-VAC), a low-cost innovation option in a developing country, designed to facilitate successful wound healing while mitigating financial limitations.

**Case presentation:**

This **case series** was reported at a tertiary referral hospital in Indonesia, involving 13 patients with soft tissue defects utilizing RAP-VAC. The average wound size diminished by 9.8 %, from 50.19 ± 36.96 cm^2^ on day 0 to 45.25 ± 36.43 cm^2^ at the endpoint measurement. The mean granulation rate was 92.13 %, and the average wound bed preparation duration was 20.92 ± 2.69 days. The whole expenditure for care expenses to IDR 2,376,923 per patient. The linear mixed model analysis significantly associated with intercept estimate of 50.19 (*p* = 0.001) with 0.32 reduction per day in the outcome (*p* = 0.010).

**Clinical discussion:**

NPWT accelerates wound shrinkage, resulting from the consistent negative pressure. The mechanical motion facilitates cell migration, hence expediting wound closure. The cost-effectiveness of NPWT has been established, diminishing the necessity for expensive therapies. A study reported using an aqua pump-based VAC, but without detailing the mechanism. In contrast, our device reverses the fan direction for improved suction. We employed the *IMITO* app measurement for objective evaluation. The RAP-VAC system, characterized by low costs, may provide substantial financial benefits while maintaining therapeutic outcomes in developing countries, as a viable choice for wound management in both hospital and community care environments.

**Conclusion:**

The findings indicate that RAP-VAC is efficacious in facilitating wound healing and serves as a cost-effective alternative to expensive VAC systems.

## Highlights

• Innovative reverse aqua pump mechanism reported in case series of reliable outcome of a modification from the traditional VAC system using a reverse aqua pump, offering a cost-effective and efficient alternative for wound management in developing country.
• Effective reduction in wound size clinical results demonstrate significant reduction in wound size, validating the reverse aqua pump as a practical innovation for promoting wound contraction and closure.
• The reverse aqua pump mechanism facilitated robust granulation tissue formation, indicating its capacity to stimulate a favorable wound healing environment comparable to conventional VAC system.
• Cost-efficiency without compromising outcomes by utilizing simplified technology, this approach markedly reduces treatment costs while maintaining effectiveness, making it accessible for broader clinical application.
• This case series provides real-world evidence supporting the reverse aqua pump VAC as a sustainable solution for managing complex wounds, particularly in healthcare systems with limited infrastructure or funding.

## 1 Introduction and importance

Soft tissue defects pose significant challenges in patient care. Conventional wound therapies are often inadequate, necessitating more effective strategies [1]. Negative Pressure Wound Therapy (NPWT), also known as Vacuum-Assisted Closure (VAC), has emerged as an effective wound management technique that promotes wound healing by removing exudate, reducing infection rate, enhancing perfusion, and stimulating tissue granulation [1,2]. Given its proven benefits, modifying VAC systems for broader use, particularly in resource-limited settings, is essential. A cost-effective innovation playing a crucial role in expanding access and improving outcomes [3].

The potential of canister-free NPWT systems can improve wound care accessibility, especially in chronic wound management. Based on this concept of cost-effective innovation, modifying NPWT systems was explored by altering consumable components such as sponges and dressings. Implemented a low-cost NPWT system using presterilized foam, plastic wrap, and feeding tubes connected to standard suction devices, achieving effective wound healing across various wound types while significantly reducing costs [4,5]. Similarly, the use of gauze in place of polyurethane foam has been proposed as a cost-effective alternative with outcomes comparable to commercial VAC systems, particularly benefiting resource-limited settings [5,6]. Numerous innovations have been introduced to reduce the cost of VAC therapy, particularly through the modification of components such as canisters, sponges, and dressings, rather than the core vacuum unit itself.

In developing countries, the high cost of commercial VAC systems limits accessibility. A study conducted in Brazil demonstrated that a low-cost NPWT system using hospital wall suction and basic dressing materials was as effective as commercial systems while significantly reducing treatment costs [7]. A randomized controlled trial in India showed that low-cost negative pressure dressings led to faster wound healing and reduced hospital visits compared to conventional dressings [8]. These studies underscore the potential of cost-effective NPWT to improve wound care in resource-limited settings.

This study assesses the **Reverse Aqua Pump Vacuum-Assisted Closure (**RAP-VAC) system in wound healing while ensuring cost-effectiveness. Attempt to reconcile therapeutic requirements with economic limitations, providing a feasible approach for the management of defect wounds. This report is presented in compliance with the Preferred Reporting of Case Series in Surgery (PROCESS) 2025 guideline [9].

## 2 Case presentation

We present a case series of patients treated with a novel, low-cost wound management approach utilizing the **RAP-VAC** system at a tertiary referral hospital in a developing country, Indonesia. The intervention was conducted between December 2023 and November 2024, following ethical approval from the institutional review board.

### 2.1 Patient selection and characteristics

Thirteen patients indicated for VAC therapy were consecutively enrolled. All patients had significant soft tissue defects, except those who had chronic wounds, diabetes, hypertension, malignancy, peripheral arterial disease, ongoing antithrombotic therapy, or refused surgical debridement were excluded. Informed consent was obtained from each patient. The case series had a **mean age of 34.2 years**, comprising **8 males (61.5** **%)** and **5 females (38.5** **%)**. Soft tissue defects were located in the **upper extremities in 4 patients (30.7** **%)** and in the **lower extremities in 9 patients (69.2** **%)**.

### 2.2 Device and protocol

All patients underwent adequate surgical debridement before initiation of VAC therapy. The **RAP-VAC device**, a locally engineered VAC system using a modified reverse aquarium aerator pump, was applied postoperatively on day 0 (H0). This device has received patent certification and distribution rights from the Indonesian Ministry of Health (Registration No. IDP000090954). **Wound assessments**, including size measurement, granulation tissue evaluation, were performed twice weekly during dressing changes. VAC therapy was continued until satisfactory granulation tissue covered the wound bed, marking the final day of treatment (HE).

**Fig. 1:**
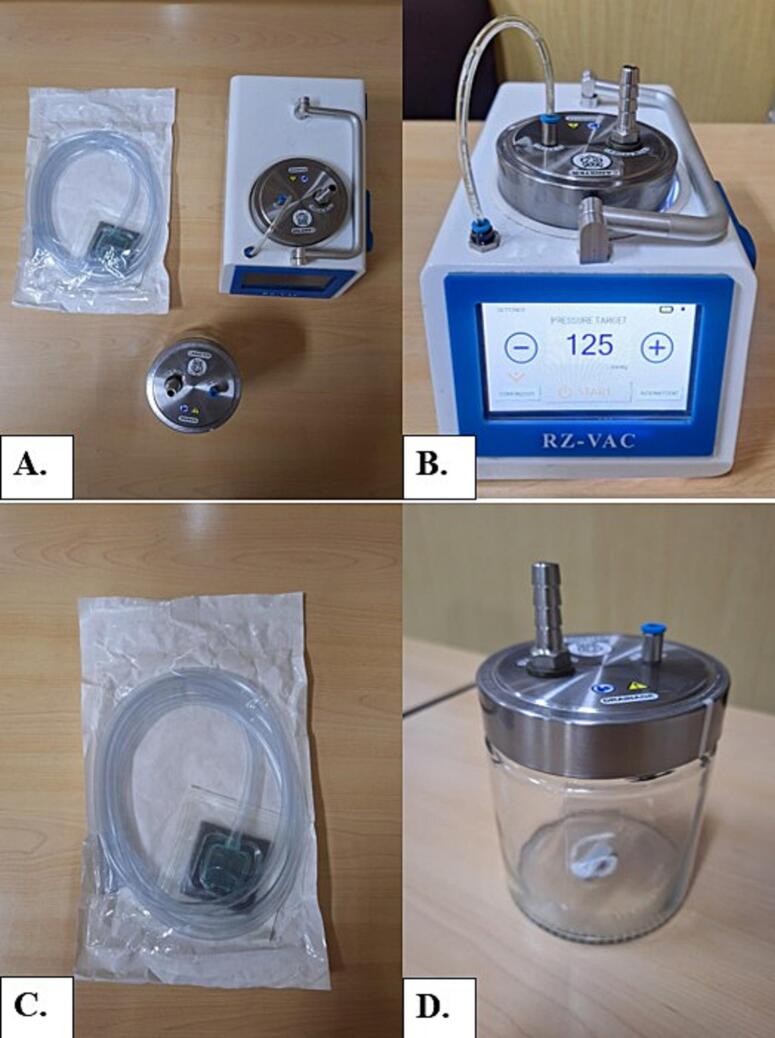
External component of the RAP VAC device (A). Touch screen interface (B). External tube (C). Reuseable canister (D).

The mechanism of the reverse aqua pump (RAP) works through coordinated components that generate and maintain negative pressure (Fig. 1). The wound exudate is collected in the Canister, which serves as the reservoir for all extracted fluid. This canister is connected via Tubing Filter to prevent overflow, backflow, and contamination that links to Reverse Aqua Motor Pump which creates sub atmospheric pressure by continuously removing air from the canister, thereby pulling wound fluid through the External Tube into the canister. The Control Panel regulates and displays the negative pressure settings to ensure therapeutic levels are maintained, monitored by Pressure Sensor. The entire setup is mounted on a Base Structure, Device Body and Back Cover. This entire system supported by an integrated Power Supply for continuous operation. The Microcontroller integrates all electronic signals, controlling the motor pump, sensors, and display to maintain optimal pressure levels. Altogether, this design functions like a “reverse aqua pump,” where instead of pushing fluid outward as in a normal pump, the system pulls fluid inward from the wound into the canister, reducing oedema, improving perfusion, and promoting granulation tissue formation.

**Fig. 2:**
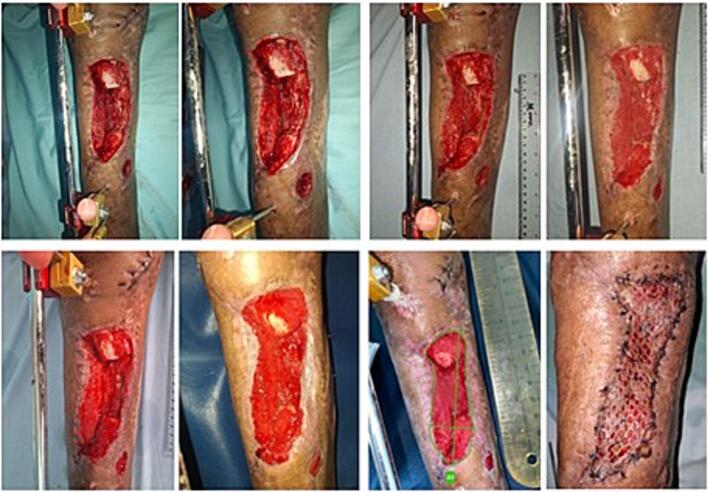
RAP-VAC application in patient post debridement.

**Fig. 3:**
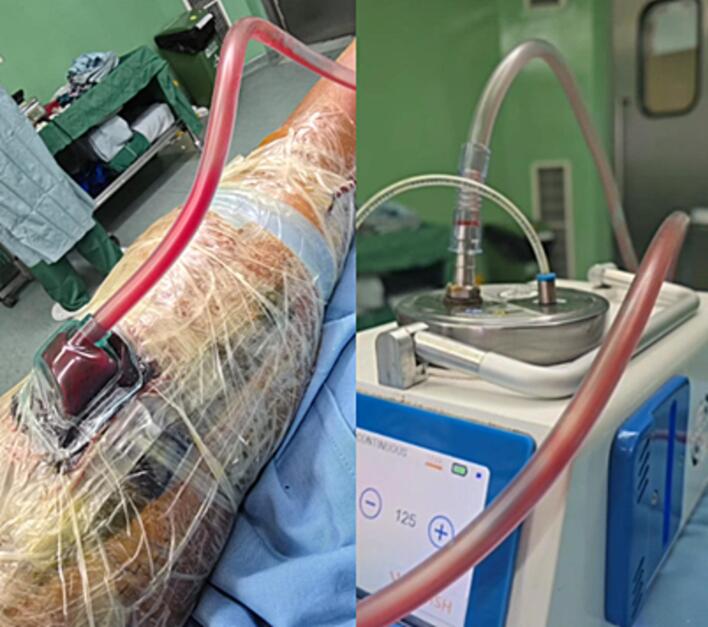
Wound bed progress documented and measure using Imito.

The protocols begin with wound debridement, followed by placement of polyurethane ether foam trimmed to wound size (Fig. 2). For deep wounds, drainage tube is layered between double layered foams, while for shallow wounds it is embedded in a single foam. The foam, tube, and surrounding skin are sealed with an occlusive drape (3 M) to ensure airtight closure, then connected to the RAP VAC device. Continuous suction at 125 mmHg is applied for high exudate, later switched to intermittent mode to promote granulation. Dressings are changed twice weekly or earlier if needed. Therapy continues until the wound bed is fully granulated, with progress documented using the IMITO application to measure wound and granulation areas (Fig. 3).

### 2.3 Clinical outcome

During the course of treatment with the RAP-VAC system, patients demonstrated notable improvements in several clinical parameters (Table 1). The average wound size at baseline (H0) was 50.19 ± 36.96 cm^2^, which decreased to 45.25 ± 36.43 cm^2^ by the endpoint (HE), reflecting an overall reduction of 9.8 %. Initially, no granulation tissue was observed (mean: 0 %), but by the end of therapy, the mean granulation rate had significantly increased to 92.12 ± 9.14 %. The average duration required for wound bed preparation, defined as the time from the initiation of VAC therapy to readiness for definitive closure, was 20.92 ± 2.69 days. In terms of economic evaluation, the cost per dressing change using the RAP-VAC system was approximately IDR 350,000, resulting in a total average cost of IDR 2,376,923 ± 384,391 per patient, relatively more affordable than commercial VAC systems. Additionally, the mean serum albumin level among patients was 3.49 ± 0.42 g/dL, indicating an overall adequate nutritional status to support the wound healing process (Table 2). The linear mixed model analysis revealed that soft tissue defect size was significantly associated with the outcome, with an intercept estimate of 50.19 (*p* = 0.001), indicating that larger initial defects corresponded to higher baseline outcome values. The wound bed preparation time demonstrated a significant reducing effect, with each additional day of preparation associated with a 0.32 reduction in the outcome (*p* = 0.010) (Table 3).

**Table 1:** Mean and standard deviation outcome of the case series.

	Mean ± SD	
Soft tissue defect size (cm)	H0	HE
	50.19 ± 36.96	45.25 ± 36.43
Tissue granulation rate/day (%)	H0	HE
	0	92.12 ± 9.14
Wound bed preparation time (day)	20.9 ± 2.69	
Cost of care (IDR)	2.376.923,08 ± 384.390.96	
Albumin (g/dL)	3.49 ± 0.42	

H0: day 0 postoperatively, HE: day endpoint therapy, IDR: Indonesian rupiah.

**Table 3:** Linear mixed-effects model estimates for defect size and preparation time.

Effect	Estimate (B)	Std. error	p-value
Intercept (soft tissue defect size)	50.19	9.77	0.001
Duration (wound bed preparation time)	−0.32	−0.063	0.010

**Table 2:** Detail result of the case series.

Case	Age	Sex	Soft tissue defect location	Soft tissue defect size (cm)		Tissue granulation rate/day		Wound bed preparation time	Cost Of care (IDR)	Albumin (g/dl)
				H0	HE	H0	HE			
1	28	Male	Left Anterior Knee	18.4	16.2	0 %	91 %	26 days	2.800.000	3.2
2	24	Female	Right Anterior Leg	31. 2	26.23	0 %	94 %	25 days	2.800.000	4.27
3	18	Female	Left Anterior Knee	32.7	11.73	0 %	92 %	21 days	2.450.000	3.23
4	4	Male	Right Anterior Posterior Thigh	4.28	4.2	0 %	88 %	18 days	2.100.000	4.2
5	18	Male	Right Dorsal Foot	83.6	82	0 %	94 %	21 days	2.450.000	2.98
6	35	Male	Left Posterior Thigh	60.4	58.2	0 %	95 %	21 days	2.450.000	3.96
7	39	Female	Left Dorsal Foot	89.9	86.5	0 %	96.6 %	18 days	2.100.000	3.63
8	58	Male	Right Anterior and Posterior Arm	97.7	91.3	0 %	97.8 %	20 days	2.450.000	3.32
9	60	Male	Right amputation stump of the right arm	11.58	10.2	0 %	100 %	18 days	2.100.000	3.4
10	67	Female	Left Posterior Elbow	5.22	5.22	0 %	86 %	21 days	2.100.000	3.42
11	42	Male	Left Anterior Thigh	40.8	31.3	0 %	98 %	18 days	2.100.000	3.68
12	27	Female	Right Anterior Knee and Lateral Thigh	65.5	63.2	0 %	65.25 %	24 days	3.200.000	2.94
13	23	Male	Right Anterior Elbow	111.2	102.06	0	100 %	21 days	1.800.000	3.23

## 3 Clinical discussion

The remarkable decrease in wound size recorded in this case series serves as a crucial indicator of the effectiveness of the RAP-VAC device. The Aqua-Pump system and standard Negative Pressure Wound Therapy (NPWT) share the same therapeutic goal of applying negative pressure to promote wound healing, but they differ in their mechanisms. The Aqua-Pump, adapted from an aquarium pump system, generates negative pressure more simply by creating continuous suction that pulls wound exudate into a canister, functioning as a low-cost reverse aqua pump. In contrast, standard NPWT devices use a more sophisticated motor pump controlled by sensors and microcontrollers to deliver consistent, adjustable negative pressure, which can be applied in continuous, intermittent, or instillation modes. This allows for more precise regulation of suction, improved control of wound environment, and optimized stimulation of granulation tissue formation. While these mechanistic differences suggest that standard NPWT may provide more reliable and reproducible outcomes compared with the Aqua-Pump, further comparative studies are needed to confirm the true clinical impact of these variations [7,8].

Notably, our VAC machine has been documented in clinical use since 2019 [10,11]. Furthermore, we have employed this device in challenging cases, including those involving mangled extremities. The result upholds the current research that emphasizes the beneficial effects of NPWT on wound healing, specifically on the shrinking of wound size. Study indicates that NPWT accelerates wound shrinkage by improving tissue perfusion and facilitating granulation tissue development [12,13]. This process primarily results from the consistent negative pressure exerted by NPWT devices, which facilitates the approximation of wound borders, thereby diminishing the surrounding area. The mechanical motion facilitates cell migration, hence expediting wound closure [14,15].

Besides the mechanical effects of negative pressure, NPWT has been demonstrated to augment angiogenesis and facilitate granulation tissue development, both of which are crucial for efficient wound repair. NPWT enhances blood circulation to the wound site, hence promoting granulation tissue production while simultaneously diminishing bacterial burden and excessive exudate [16]. The role of NPWT in neovascularization, which improves blood perfusion and expedites re-epithelialization. The interplay of these actions leads to a notable decrease in wound size throughout the treatment duration, as supported by the findings of the present investigation [17]. The presence of exposed tissue and small open vessels theoretically increases the risk of blood loss, especially during the initial postoperative period. However, most studies have shown that when negative pressure settings are appropriately adjusted often starting at the lower end of the recommended range (e.g., 75–125 mmHg) and hemostasis is adequately achieved before VAC application, the risk of clinically significant bleeding remains low. Moreover, current guidelines generally recommend ensuring meticulous hemostasis before initiating negative pressure wound therapy (NPWT), and several clinical reports have documented its safe use even on day 0 post-debridement when these precautions are followed [1,14].

This case series revealed a remarkable wound granulation rate of 92.12 ± 9.14 %, highlighting the RAP-VAC system's efficacy in facilitating successful wound repair. Granulation tissue is essential for wound healing, serving as a framework for tissue development and indicating the shift from the inflammatory to the proliferative phase of healing [18]. The RAP-VAC system functions in promoting granulation tissue formation, aligns with previous research that has established corresponding results with NPWT, underscoring the significance of mechanisms like fluid instillation and exudate management in facilitating granulation tissue development [12,13]. Additionally, the consistent assessment of granulation tissue during each dressing change is essential for facilitating rapid treatment when required, hence enhancing general repair results [19].

Wound bed preparation, as used in this case series, refers to the duration necessary for the wound to attain a state conducive to definitive closure and was an additional critical result. The mean duration for wound bed preparation was 20.92 ± 2.69 days, aligning with recent research that underscores the significance of prompt wound bed preparation in enhancing healing results [19]. Efficient wound bed preparation is essential for minimizing the possibility of infection, eliminating necrotic tissue, and creating a perfect environment for healing. The RAP-VAC system enhances these processes by encouraging wound debridement, diminishing bacterial burden, and regulating exudate, which are crucial for establishing an optimal healing process [12,13].

The average duration for wound bed preparation via NPWT was 18.7 days, implying that NPWT can facilitate a preparedness for surgical treatments, including split-thickness skin grafting [20]. This duration aligns with the established notion that NPWT facilitates granulation tissue development and diminishes bacterial burden, both essential for optimizing the wound bed for closure [21]. Furthermore, NPWT is especially advantageous for extensive or intricate wounds, where prompt wound bed preparation is essential for the efficacy of surgical reconstruction [6]. The results of this research uphold previous observations, indicating that RAP-VAC markedly decreases the duration necessary for wound bed preparation relative to conventional approaches.

A crucial element of wound care is the expense of treatment, especially in resource-constrained environments. The mean expense for each dressing change utilizing the RAP-VAC system was determined to be IDR 350,000, culminating in an overall treatment expenditure of IDR 2,376,923 per patient. Cost-effectiveness is particularly pertinent given the escalating healthcare expenses and the demand for economical wound management options [19]. Numerous studies have underscored the financial strain of conventional NPWT systems, with certain reports indicating expenses ranging from $200 to $500 every dressing change, which can rapidly escalate throughout the treatment duration [22]. Conversely, the RAP-VAC system's comparatively low cost presents a feasible option, especially in environments where financial limitations provide a substantial obstacle to care.

Prior studies have shown that NPWT, although initially more expensive, can decrease total treatment costs by reducing hospital durations and lessening the necessity for pricier procedures [19,23]. The cost-effectiveness of NPWT has been established in surgical contexts, demonstrating its ability to avert complications and diminish the necessity for expensive therapies [23]. A study reported using an aqua pump-based VAC, but without detailing the mechanism. In contrast, our device reverses the fan direction for improved suction [24]. While their wound assessment used subjective terms (*Better*, *Same*, *Worse*), we employed the *Imito* app measurement for objective evaluation. Both studies support the effectiveness of aqua pump-based VAC therapy. The RAP-VAC system, characterized by reduced costs, may provide substantial financial benefits while maintaining therapeutic outcomes, rendering it a viable choice for wound management in both hospital and community care environments.

## 4 Conclusion

In conclusion, the RAP-VAC system demonstrated significant benefits in wound healing, including substantial reductions in wound size, enhanced granulation tissue formation, and timely wound bed preparation. These results align with an affordable and effective alternative to more expensive VAC systems. The lack of a control group limits the ability to generalize the findings to a broader population. Future study on further evaluating the cost-effectiveness of RAP-VAC in larger cohorts and exploring its potential for use in diverse clinical settings.

## Ethical approval

This research has received approval from the Ethics Committee of the Faculty of Medicine, Universitas Gadjah Mada (UGM), Yogyakarta, with ethical clearance approval letter number KE/FK/0570/EC/2024.

## Guarantor

M.R.Z.

## Funding

This research received no external funding.

## Consent for publications

Written informed consent was obtained from the patient for publication of this case report and any accompanying images. A copy of the written consent is available for review by the Editor-in-Chief of this journal.

## CRediT authorship contribution statement

**Meirizal:** Conceptualization, Writing – original draft, Validation, Investigation. **Rahadyan Magetsari:** Validation, Methodology, Writing – review & editing, Supervision. **Mohammad Rizal Chaidir:** Visualization, Resources, Methodology, Supervision. **Sumadi Lukman Anwar:** Writing – original draft, Resources, Validation. **Agung Susilo Lo:** Resources, Writing – original draft, Visualization, Validation. **I. Made Dolly:** Validation, Methodology, Writing – review & editing, Supervision.

## Declaration of competing interest

The authors declare no conflict of interest.

## Data Availability

Supporting data will be available upon reasonable request.
